# Long-term effects of stimulants on neurocognitive performance of Taiwanese children with attention-deficit/hyperactivity disorder

**DOI:** 10.1186/1471-244X-13-330

**Published:** 2013-12-04

**Authors:** Ching-Shu Tsai, Yu-Shu Huang, Chen-Long Wu, Fang-Ming Hwang, Kin-Bao Young, Ming-Horng Tsai, Shih-Ming Chu

**Affiliations:** 1 Department of Psychiatry, Chang Gung Memorial Hospital and University, Chiayi, Taiwan; 2 Department of Child Psychiatry, Chang Gung Memorial Hospital and University, Linkou, Taiwan; 3 Department of Occupational and Environmental Medicine, National Cheng Kung University Hospital, Tainan, Taiwan; 4 Department of Environmental and Occupational Health, College of Medicine, National Cheng Kung University, Tainan, Taiwan; 5 Department of Education, National Chia-Yi University, Chiayi, Taiwan; 6 Infant and Child Care Department, National Taipei University of Nursing and Health Science, Taipei, Taiwan; 7 Department of Pediatrics, Division of Pediatric Neonatology, Chang Gung Memorial Hospital, Taoyuan, Taiwan

**Keywords:** Attention-deficit/hyperactivity disorder, Taiwan, Intelligence, WISC-III, IQ

## Abstract

**Background:**

Attention-deficit/hyperactivity disorder (ADHD) is a common behavioral and neurocognitive disorder in school-age children. Methylphenidate (MPH) is the most frequently prescribed CNS stimulant for ADHD. The aim of this study is to evaluate the changes in intelligence quotient and domains of neurocognitive function after long-term MPH treatment of Taiwanese children with ADHD.

**Methods:**

The Wechsler Intelligence Scale (WISC-III) was administrated twice at an interval of at least one year for all 171 subjects (6–12 years) and 47 age- and gender-matched children without ADHD. The ADHD-Rating scale and Clinical Global Impression-Severity (CGI-S) were also used at the time of enrolment, and at 6 months and one year later.

**Results:**

Taiwanese children with ADHD had lower Verbal IQ (VIQ) and Full IQ (FIQ) and performed poorly on several subtests of the WISC-III, including Similarities, Vocabulary, and Coding, compared to healthy children without ADHD. After one year of MPH treatment, significant decrements in all scores of the ADHD-Rating scale and CGI-S and increments in several domains of the WISC-III, including FIQ, VIQ, PIQ, Perceptual Organization Index (POI), Picture Completion, Picture Arrangement, Object Assembly, and Digit Span were observed. When the ADHD children under MPH treatment were subdivided into two age groups (6–8 years and 9–12 years), significantly better performance in some subtests and subscales of the WISC-III (such as Similarities, Comprehension, and Object assembly) was found in the 6–8 years age group.

**Conclusions:**

Long-term MPH treatment may improve the neurocognitive profiles of the ADHD children, as seen in their performance in several subtests and in the IQ scores on the WISC-III. And this improvement had no correlation with the decrement of ADHD symptoms. Starting stimulant treatment at as young an age as possible is advised due to the greater benefits in the 6–8 years age group, as seen in this study. More research in this area is also needed to confirm these results.

## Background

Attention deficit hyperactivity (ADHD), one of the most common behavioral and neurocognitive disorders [1], is characterized by persistent over activity, inattention and impulsivity. The American Psychiatric Association (APA, 1994) diagnostic guideline [Diagnostic and Statistical Manual, version IV (DSM-IV)] classified ADHD into three subtypes: (a) inattentive, (b) hyperactive–impulsive and (c) combined. According to the DSM-IV, text version (DSM-IV-TR) diagnostic guidelines, the prevalence of ADHD is estimated at 3–7% in school-age children [2,3]. Due to the nature of the populations sampled, diagnostic criteria used, cultural differences, and methodological limitations, the prevalence of ADHD in various cultures varies. The prevalence is estimated to be about 8.4–11.7% in Taiwan [4]; 2.4% in Australia [5]; and 4% in Japan [6]. The negative outcomes of ADHD include peer rejection, low self-esteem, academic underachievement, and learning disabilities [7,8]. Cognitive function impairment as in executive dysfunction and a lower intellectual coefficient were also found in ADHD children. Although the concept of executive function is broad and variable, overall it is used in reference to the higher brain process involved in planning problem-solving and can be screened using the Wechsler Intelligence Scale (WISC) [9]. From the intellectual coefficient perspective, a meta-analytic review by Frazier et al. reported that the ADHD groups performed at an average of 9.15 points lower than the control group in their Full Scale Intelligence Quotient (FSIQ), assuming a theoretical standard deviation of 15 [10].

Methylphenidate (MPH), a kind of central nervous system (CNS) stimulant, is the most frequently prescribed medication for ADHD, and its clinical efficacy is well established [11]. The effects of MPH on attention result from a combination of noradrenergic and dopaminergic mechanisms [12], which are assumed to ameliorate the two principal problems of attention deficiency and impulsivity/hyperactivity in children with ADHD. Moreover, scholars hypothesized that treating ADHD symptoms via stimulant drugs not only decreased the negative impact of ADHD, but possibly also benefited the neurocognitive performances of children with ADHD [13]. A previous meta-analysis by Kavale et al. found that stimulant treatment for an average period of 18 weeks led to a mean gain of about 6 to 7 points in FSIQ, and 6 points in both the Verbal Intelligence Quotient (VIQ) and Performance Intelligence Quotient (PIQ) [14]. Another meta-analysis by Thurber and Walker [15] also demonstrated that short-term stimulant drug effects could result in a mean increase of 2.25 points in the FSIQ. Recent studies that focus on intellectual outcomes after a long-term treatment period have shown that long-term stimulant treatment for at least one year could increase the Intelligence Quotient (IQ) score in children with ADHD, and that more improvement in IQ scores was found in the group of those who could maintain a longer treatment duration [16,17]. That is, the intellectual performance of children with ADHD may be improved with long-term CNS stimulant treatment, as well as short-term stimulant treatment, but a bigger gain with long-term treatment is suspected. In addition to the decreased severity of core symptoms, avoidance of negative consequences and improved neurocognitive performances of children with ADHD, MPH has been shown to counteract the detrimental effects of earlier-origin developmental insults [18] and has age-dependent effects on prefrontal neurons [19,20]. So it is also suspected that starting drug treatment at an earlier age may have more benefits.

In Taiwan, a high prevalence rate of ADHD was noticed about ten years ago [4], but there is still little research comparing neurocognitive function between children with ADHD and healthy children. And even though the positive effect of CNS stimulants in ADHD treatment has been reported in many studies, there are still few studies examining the long-term effect of CNS stimulants on neurocognitive function and in different age groups. Therefore, in this study, we have several objectives as below: 1) to better understand the neurocognitive performance of ADHD children in Taiwan as measured by the WISC-III and to further compare the results with healthy controls; 2) to investigate the changes in rating scale and IQ and the influences on the domains of the WISC subtests after long-term MPH treatment in Taiwanese children with ADHD; 3) to explore if there is more benefit to long-term MPH treatment; and 4) to know if MPH treatment has a diverse effect on neurocognitive profiles in different age groups.

## Methods

### Participants

We collected ADHD children from the outpatient clinics of a medical center in northern Taiwan from December 2005 to 2006. Inclusion criteria were: age 6–12 years; and a clinical diagnosis of ADHD based on the relevant DSM-IV diagnostic criteria and standardized clinical assessments, as determined by experienced board-certificated child psychiatrists. Study approval was obtained from the Institutional Review Board of Chang Gung Memorial Hospital. Written informed consent was obtained from the parents of each child and children following an explanation of the study.

The diagnostic assessment of ADHD were performed by two senior child psychiatrists, who administered structured interviews using the Chinese-language version of the Schedule for Affective Disorder and Schizophrenia for School-Age Children [21], epidemiologic version (K-SADS-E) [22]. This evaluation affirmed the clinical diagnosis and excluded children who had co-morbid pervasive developmental disorder, or a history of bipolar disorder, psychosis, anxiety disorder, seizure disorder, substance abuse or mental retardation. Other physical and neurological examinations were performed by a pediatrician and neurologist to rule out CNS or other physical diseases. All ADHD children enrolled in this study were drug-naïve at baseline. Treatment involved administering 0.3-7 mg/kg/dose of MPH to all subjects, which was within the range of the average daily dose in Taiwan [23].

### Measurements

Since executive function could be screened by the WISC [9], we chose this psychometric measurement to evaluate the neurocognitive performance of ADHD and healthy children. After the initial screening, all the eligible children were administered the WISC-III [24] by experienced child psychologists. The ADHD-Rating scale [25] and Clinical Global Impression-Severity (CGI-S) [26] were also scored simultaneously by an experienced child psychiatrist.

The WISC-III is a standardized intelligence test suitable for children and adolescents aged 6–16 years. It contains 13 subtests, namely Information, Similarities, Arithmetic, Vocabulary, Comprehension, Digit Span, Picture Completion, Coding, Picture Arrangement, Block Design, Object Assembly, Symbol Search and Maze. VIQ, PIQ and full IQ (FIQ), and their scores can be calculated based on these subtests. The verbal comprehension index (VCI), perceptual organization index (POI), freedom from distractibility index (FDI), and processing speed index (PSI) were also calculated from these subtests. The WISC-III was used when the participants first enrolled in the study. Repeated WISC-III tests were administered for those who stayed in the study and received CNS stimulant treatment for one year. The second WISC-III test was administered at least one year from the first test. All participants were free from medication for at least one week prior to the first and the second administration of the WISC-III.

The ADHD-Rating scale is a structured interview completed by the investigator based on information from the parents and child. Each of the items has a 4-point response scale, and each item explores one of the 18 criteria outlined in the DSM-IV to diagnose ADHD. Higher scores indicate greater severity of ADHD. Initial evaluation was scored when the ADHD children were enrolled, and again at 6 months and one year into treatment.

The CGI-S uses a 7-point scale with responses ranging from 1 (normal) to 7 (the most severely ill patients). The scale was filled out at initial intake, and at 6 months and one year into the study, coinciding with the administration of the ADHD-Rating scale.

### Control group

To compare the differences between healthy children without ADHD and children with ADHD in their performance on the intelligence test, we recruited 47 age-matched children (6–12 years) from surrounding schools in the community by contacting the teachers. Inclusion criteria included healthy children, as reported by the parents, and the absence of ADHD symptoms. All children in the control group were evaluated at the “child developmental evaluation center” of the hospital, where physical, neurological and mental evaluations were performed by the same specialists that saw the suspected ADHD children. The exclusion criteria for the control group were the same as those for the ADHD group.

### Data analysis

The variables of sample characteristics, scores on the ADHD-Rating scale and CGI-S, and profiles of neurocognitve performance evaluated via the WISC-III between the healthy normal controls and the ADHD children were compared using two sample t-tests and the chi-square test. The changes in the ADHD-rating scale and CGI-S and in performances on the WISC-III before and after MPH treatment in the ADHD group were compared using the paired t-test. The correlation between the changes in the severity of ADHD symptoms and performance on the WISC-III was analyzed via Pearson's correlation coefficient. All tests were performed using SPSS 15.0, with a 2-tailed significance level of 0.05.

## Results

A total of 171 children with ADHD were included in our study.

### Differences in the neurocognitive performance of ADHD and healthy normal children

Compared to the healthy children, the ADHD children had significant differences in their scores on the ADHD-Rating scale (Table 1), including inattention (p < 0.001), hyperactivity (p < 0.001) and total (p < 0.001) scores, and on the CGI-S (p < 0.001). Children with ADHD also scored significantly worse than children in the control group in FIQ (p < 0.05), VIQ (p < 0.05) and several subtests of the WISC-III, including Similarities (p < 0.01), Vocabulary (p < 0.05), Coding (p < 0.05) and VCI (p < 0.05).

**Table 1:** Characteristics of ADHD and normal children

	**ADHD (N = 171)**	**Control (N = 47)**	**p value**^**1**^	
	**Mean ± SD or %**	**Mean ± SD or %**		
Age	8.7 ± 1.8	8.8 ± 2.1	0.654	
Male	81.9%	51.1%	<0.001	***
BMI	18.5 ± 3.7	18.8 ± 3.3	0.472	
ADHDRS_total	29.1 ± 8.2	9.8 ± 6.5	<0.001	***
ADHDRS_inattention	16.5 ± 4.2	6.7 ± 2.9	<0.001	***
ADHDRS_hyperactivity	12.8 ± 5.1	3.2 ± 4.0	<0.001	***
CGI –S	4.5 ± 0.8	1.0 ± 0.8	<0.001	***
**WISC-III**				
Information	9.3 ± 2.9	10.3 ± 2.7	0.182	
Similarities	9.8 ± 3.3	12.4 ± 2.5	0.003	**
Arithmetic	10.2 ± 3.1	11.4 ± 1.8	0.121	
Vocabulary	9.6 ± 3.1	11.3 ± 2.5	0.032	*
Comprehension	9.7 ± 2.8	11.0 ± 2.4	0.087	
Picture completion	9.7 ± 3.0	10.9 ± 2.0	0.054	
Coding	9.1 ± 3.6	11.1 ± 3.6	0.033	*
Picture arrangement	9.7 ± 2.9	11.7 ± 2.9	0.009	
Block design	10.5 ± 3.5	11.4 ± 2.3	0.158	
Object assembly	9.2 ± 3.1	9.9 ± 3.3	0.407	
Digit span	9.5 ± 3.0	-		
Symbol search	10.5 ± 3.1	-		
FIQ	97.6 ± 14.3	106.6 ± 9.5	0.014	*
VIQ	98.2 ± 13.9	107.0 ± 11.0	0.020	*
PIQ	98.2 ± 15.5	104.9 ± 9.6	0.103	
VCI	99.0 ± 13.6	107.8 ± 11.6	0.017	*
POI	98.6 ± 14.4	105.4 ± 10.7	0.078	
FDI	98.7 ± 15.0			
PSI	99.1 ± 15.5			

^1^two sample t test, *: <0.05, **: <0.01 and ***: <0.001.

FIQ = full intelligence quotient, VIQ = verbal intelligence quotient, PIQ = performance intelligence quotient, VCI = verbal comprehension index, POI = perceptual organization index, FDI = freedom from distractibility index, PSI = processing speed index.

### Differences between ADHD children who received stimulant drugs or not

Of the 171 ADHD children, 130 received MPH treatment. The 41 ADHD children without MPH treatment were younger (p < 0.01) and had less severity on the ADHD-Rating scale, including hyperactivity (p < 0.01) and total (p < 0.05) scores and on the CGI-S (p < 0.01), than those under MPH treatment. However, there was no significant difference in performance on the WISC-III between these two groups.

### Difference within drug treatment groups between those who received MPH beyond one year and those who were lost to follow-up

In the MPH treatment group, 103 participants (79%) continued in the study beyond one year. Children who were lost to follow-up during the period of MPH treatment had the same severity of ADHD symptoms, but had better performance in FIQ (p < 0.05), VIQ (p < 0.05), PIQ (p < 0.05), Arithmetic (p < 0.01), and Vocabulary (p < 0.001).

### Changes in neurocognitive function after long-term MPH treatment

The 103 participants who stayed in the study beyond one year were administered the ADHD-Rating scale and the CGI-S at 6 months and one year. Psychometric measures using the WISC-III were also completed for the 103 participants again at the end of one year. All subscores on the ADHD-Rating scale, including inattention (p < 0.001), hyperactivity (p < 0.001) and total (p < 0.001), and the CGI-S (p < 0.001) decreased significantly for ADHD children after drug therapy. Significant increments in the FIQ (p < 0.001), VIQ (p < 0.05) and PIQ (p < 0.001) scores were also found in the MPH treatment group. Among the subtests of the WISC-III, significant improvements in performance were found in Picture Completion (p < 0.01), Picture Arrangement (p < 0.01), Object Assembly (p < 0.05), and Digit Span (p < 0.05). The POI (p < 0.001) score also improved significantly after drug therapy (Table 2).

**Table 2:** Changes in ADHD symptoms and WISC-III scores of ADHD children after MPH treatment for one year; N = 103

	**Mean ± SD**	**P value**^**1**^	
ADHDRS_total	−7.8 ± 6.7	<0.001	***
ADHDRS_inattention	−4.0 ± 3.8	<0.001	***
ADHDRS_hyperactivity	−3.8 ± 3.7	<0.001	***
CGI –S	−0.9 ± 0.8	<0.001	***
**WISC-III**			
Information	0.3 ± 1.6	0.103	
Similarities	0.4 ± 2.0	0.075	
Arithmetic	0.1 ± 1.9	0.504	
Vocabulary	0.3 ± 1.9	0.074	
Comprehension	−0.1 ± 1.6	0.434	
Picture completion	0.8 ± 2.0	0.001	**
Coding	0.0 ± 2.7	0.911	
Picture arrangement	0.8 ± 1.9	0.001	**
Block design	0.2 ± 2.0	0.212	
Object assembly	0.6 ± 2.1	0.012	*
Digit span	0.6 ± 1.9	0.043	*
Symbol search	−0.6 ± 2.1	0.090	
FIQ	2.9 ± 7.5	<0.001	***
VIQ	1.6 ± 7.1	0.045	*
PIQ	3.9 ± 9.0	<0.001	***
VCI	0.7 ± 6.1	0.312	
POI	3.4 ± 7.3	<0.001	***
FDI	1.0 ± 6.8	0.324	
PSI	0.2 ± 9.4	0.874	

^1^paired t test, *: <0.05, **: <0.01 and ***: <0.001.

### Differences in the changes in neurocognitive function after long-term MPH treatment among the ADHD children with or without learning disorders

ADHD is often comorbid with learning disorders (LD), which would influence performance on the WISC-III. We subdivided the ADHD children with MPH treatment into two groups. One group included 19 ADHD children comorbid with LD and the other, ADHD children without LD. However, we did not find a significant difference between the two groups in the changes in ADHD symptoms and in the WISC-III after MPH treatment.

### Changes in neurocognitive function of ADHD children with/without MPH treatment at the end of one year

We compared the differences between ADHD children with/without MPH treatment for one year. The results showed that there was a significant increase in FIQ (p < 0.001), VIQ (p < 0.05), PIQ (p < 0.001), Picture completion (p < 0.001), Picture arrangement (p < 0.01), Object assembly (p < 0.05), Digit span (p < 0.05) and POI (p < 0.001) among the ADHD children who received MPH treatment. However, there were no significant changes in performance on the WISC-III among the ADHD children not receiving MPH treatment. The sample size of the ADHD children who received and did not receive MPH treatment was 103 and 17, respectively.

### Effect of MPH treatment on neurocognitive profiles in different age groups

The ADHD children under MPH treatment were subdivided into two age groups (6–8 years and 9–12 years). The pre-treatment data of the 6–8 years age group were found to be significantly higher in inattention (p < 0.01), hyperactivity (p < 0.05), and total scores (p < 0.01) on the ADHD-rating scale and CGI-S (p < 0.01), and significantly lower on the Similarities subtest of the WISC-III (p < 0.01) (Table 3). After one year of MPH treatment, there were significant decrements in severity of ADHD symptoms in both age groups, but without a statistically significant difference in the range of improvement of the two groups (Table 4). Significant improvements in Similarities (p < 0.05), Picture Completion (p < 0.05), Object Assembly (p < 0.01), FIQ (p < 0.01), and POI (p < 0.05) were noted in the 6–8 years age group, and significant improvements in Picture Completion (p < 0.05), Picture Arrangement (p < 0.01), FIQ (p < 0.05), PIQ (p < 0.01) and POI (p < 0.01) were noted in the 9–12 years age group. However, the Comprehension subtest score decreased significantly in the 9–12 years age group (p < 0.05) after MPH treatment. When comparing the range of changes in the WISC-III across these two age groups, more improvement with statistical significance in the performances of Similarities (p < 0.05), Comprehension (p < 0.05) and Object Assembly (p < 0.05) were found in 6–8 years age group after one year of MPH treatment. Based on these results, more benefit from receiving MPH treatment could be found in the younger age group (6–8 years) than in the older age group (9–12 years).

**Table 3:** Pretreatment characteristics of two age groups of ADHD children

	**6-8 years (N = 60)**	**9-12 years (N = 70)**	**P value**^**1**^	
**Item**	**Mean ± SD**	**Mean ± SD**		
ADHDRS_total	32.2 ± 8.0	27.9 ± 7.7	0.006	**
ADHDRS_inattention	17.8 ± 5.1	15.7 ± 3.3	0.007	**
ADHDRS_hyperactivity	14.4 ± 5.1	12.6 ± 4.7	0.017	*
CGI –S	4.8 ± 0.8	4.4 ± 0.9	0.007	**
**WISC-III**				
Information	9.4 ± 2.4	9.3 ± 3.2	0.736	
Similarities	8.8 ± 3.4	10.7 ± 3.1	0.002	**
Arithmetic	10.0 ± 3.2	10.3 ± 3.1	0.620	
Vocabulary	9.4 ± 3.5	9.9 ± 2.5	0.433	
Comprehension	9.8 ± 3.1	9.7 ± 2.5	0.830	
Picture completion	9.2 ± 2.9	9.9 ± 3.2	0.211	
Coding	9.2 ± 4.2	8.9 ± 3.2	0.715	
Picture arrangement	9.6 ± 2.8	9.8 ± 3.1	0.673	
Block design	10.5 ± 3.1	10.8 ± 3.5	0.625	
Object assembly	9.0 ± 2.7	9.5 ± 3.2	0.414	
Digit span	9.3 ± 2.6	9.8 ± 3.2	0.481	
Symbol search	11.0 ± 2.7	11.0 ± 2.4	0.989	
FIQ	96.4 ± 13.7	99.0 ± 14.6	0.298	
VIQ	97.1 ± 14.8	99.5 ± 13.4	0.369	
PIQ	97.8 ± 13.8	98.9 ± 16.5	0.720	
VCI	97.3 ± 13.8	100.7 ± 13.2	0.181	
POI	97.2 ± 12.6	100.2 ± 15.1	0.273	
FDI	98.5 ± 13.0	100.8 ± 15.8	0.504	
PSI	100.6 ± 16.7	99.9 ± 12.7	0.827	

^1^P value by 2 sample t-test; *: <0.05, **: <0.01.

**Table 4:** Changes in ADHD symptoms and WISC-III scores in the two age groups after MPH treatment for one year

	**6-8 years (N = 47)**		**9-12 years (N = 56)**		**P value**^**2**^	
**Item**	**Mean ± SD**	**Sig.**^**1**^	**Mean ± SD**	**Sig.**^**1**^		
ADHDRS_total	−7.0 ± 6.9	***	−8.4 ± 6.6	***	0.351	
ADHDRS_inattention	−3.6 ± 4.0	***	−4.3 ± 3.5	***	0.332	
ADHDRS_hyperactivity	−3.5 ± 3.6	***	−4.0 ± 3.8	***	0.526	
CGI –S	−0.9 ± 0.7	***	−0.9 ± 1.0	***	0.892	
**WISC-III**						
Information	0.4 ± 1.6		0.2 ± 1.6		0.695	
Similarities	1.1 ± 2.4	*	−0.1 ± 1.4		0.013	*
Arithmetic	0.1 ± 2.4		0.1 ± 1.5		0.996	
Vocabulary	0.5 ± 2.2		0.2 ± 1.6		0.545	
Comprehension	0.4 ± 1.7		−0.5 ± 1.5	*	0.015	*
Picture completion	0.7 ± 2.0	*	0.9 ± 2.1	*	0.753	
Coding	−0.4 ± 3.0		0.4 ± 2.3		0.093	
Picture arrangement	0.5 ± 1.7		1.0 ± 2.0	**	0.254	
Block design	0.3 ± 2.1		0.2 ± 1.9		0.962	
Object assembly	1.2 ± 2.0	**	0.2 ± 2.1		0.048	*
Digit span	0.7 ± 2.0		0.5 ± 1.9		0.770	
Symbol search	−1.1 ± 2.8		−0.2 ± 1.7		0.190	
FIQ	3.6 ± 7.0	**	2.3 ± 7.8	*	0.367	
VIQ	2.6 ± 8.5		0.8 ± 5.7		0.280	
PIQ	2.8 ± 8.8		4.9 ± 9.2	**	0.315	
VCI	2.2 ± 6.3		−0.5 ± 5.8		0.066	
POI	3.4 ± 7.6	*	3.4 ± 7.2	**	0.992	
FDI	0.3 ± 6.6		1.4 ± 7.0		0.616	
PSI	−1.3 ± 11.8		1.1 ± 7.8		0.421	

^1^by paired t-test; ^2^P value by 2 sample t-test.

*: <0.05, **: <0.01 and ***: <0.001.

### Effect of MPH treatment on neurocognitive profiles in different age groups of ADHD children with or without LD

When further subdividing the group of ADHD children under MPH treatment comorbid with or without LD into two age groups (6–8 years and 9–12 years), similar results could be noted. More improvement with statistical significance in the performances of Similarities (p < 0.01), Comprehension (p < 0.05) and Object Assembly (p < 0.05) was found in 6–8 years age group.

### Correlation between the changes in neurocognitive profiles and severity of ADHD symptoms

No significant correlation was found between the changes in performance on the WISC-III (including 13 subtests, VCI, POI, FDI, PSI, FIQ, VIQ, and PIQ) and the severity of ADHD symptoms as measured by the ADHD-Rating scale and CGI-S (Table 5).

**Table 5:** Pearson correlation coefficients (R) between the changes in performance on the WISC-III and the changes of ADHD symptoms and CGI-S

**WISC-III**	**ADHD RS_total**		**ADHD RS_inattention**		**ADHD RS _hyperactivity**		**CGI-S**	
	**R**	**P value**	**R**	**P value**	**R**	**P value**	**R**	**P value**
Information	0.022	0.864	0.009	0.943	0.032	0.806	0.136	0.299
Similarities	0.177	0.169	0.133	0.303	0.193	0.129	0.152	0.241
Arithmetic	0.001	0.995	−0.091	0.409	0.096	0.378	−0.024	0.826
Vocabulary	0.004	0.974	0.076	0.485	−0.071	0.510	0.020	0.856
Comprehension	−0.003	0.982	−0.023	0.856	0.013	0.922	−0.224	0.083
Picture completion	0.229	0.076	0.168	0.195	0.237	0.063	0.132	0.315
Coding	−0.016	0.883	−0.060	0.583	0.026	0.814	0.074	0.503
Picture arrangement	0.072	0.577	0.040	0.757	0.087	0.499	0.043	0.745
Block design	0.018	0.870	−0.014	0.897	0.048	0.656	0.008	0.944
Object assembly	0.104	0.419	0.094	0.469	0.091	0.479	0.160	0.223
Digit span	−0.176	0.272	−0.164	0.305	−0.090	0.571	−0.123	0.448
Symbol search	−0.032	0.853	−0.177	0.303	0.107	0.527	−0.120	0.494
FIQ	0.081	0.459	0.032	0.768	0.117	0.284	0.017	0.877
VIQ	0.053	0.676	0.022	0.864	0.078	0.531	0.083	0.512
PIQ	0.104	0.407	0.065	0.604	0.115	0.354	0.154	0.219
VCI	0.122	0.356	0.135	0.310	0.090	0.492	0.010	0.942
POI	0.234	0.074	0.178	0.176	0.236	0.070	0.228	0.085
FDI	−0.186	0.257	−0.300	0.063	−0.010	0.950	−0.106	0.528
PSI	0.035	0.841	0.009	0.957	0.045	0.791	0.195	0.263

ADHD RS: attention-deficit/hyperactivity disorder rating scale; CGI-S: clinical global impression-severity; R: Pearson’s correlation coefficients.

### Correlation between the changes in neurocognitive profiles and baseline severity of ADHD symptoms

Similarly, there were no significant associations between the changes of WISC-III and the baseline ADHD severity and CGI scores in the MPH treatment group (Table 6).

**Table 6:** Pearson correlation coefficients (R) between the changes in performance on the WISC-III and the baseline ADHD symptoms and CGI-S

**WISC-III**	**ADHDRS _total**		**ADHDRS _inattention**		**ADHDRS _hyperactivity**		**CGI-S**	
	**R**	**P value**	**R**	**P value**	**R**	**P value**	**R**	**P value**
Information	−0.040	0.744	0.047	0.701	−0.111	0.359	−0.138	0.254
Similarities	−0.040	0.737	−0.015	0.900	−0.008	0.949	−0.016	0.893
Arithmetic	−0.068	0.510	−0.039	0.703	−0.029	0.778	0.083	0.418
Vocabulary	−0.116	0.255	−0.032	0.752	−0.129	0.205	−0.077	0.451
Comprehension	−0.001	0.997	−0.213	0.072	0.084	0.481	0.223	0.060
Picture completion	−0.157	0.195	−0.105	0.387	−0.016	0.893	−0.068	0.574
Coding	−0.109	0.288	−0.119	0.248	0.026	0.802	−0.130	0.203
Picture arrangement	−0.180	0.135	−0.107	0.379	−0.133	0.272	−0.114	0.346
Block design	−0.099	0.330	−0.043	0.677	−0.133	0.193	−0.086	0.400
Object assembly	−0.015	0.901	0.044	0.715	−0.024	0.841	−0.073	0.550
Digit span	0.146	0.329	0.222	0.134	0.047	0.754	0.138	0.354
Symbol search	0.131	0.409	0.143	0.367	0.075	0.638	0.205	0.192
FIQ	−0.112	0.274	−0.080	0.435	−0.060	0.561	0.000	0.998
VIQ	−0.156	0.184	−0.125	0.289	−0.138	0.242	−0.045	0.704
PIQ	−0.177	0.128	−0.098	0.402	−0.089	0.447	−0.164	0.160
VCI	−0.105	0.391	−0.091	0.456	−0.089	0.467	0.000	0.998
POI	−0.175	0.151	−0.079	0.521	−0.114	0.349	−0.159	0.193
FDI	−0.091	0.551	0.062	0.684	−0.152	0.319	0.030	0.843
PSI	−0.105	0.507	−0.016	0.920	0.003	0.983	−0.127	0.422

ADHD RS: attention-deficit/hyperactivity disorder rating scale; CGI-S: clinical global impression-severity; R: Pearson’s correlation coefficients.

## Discussion

In this study, children with ADHD had lower VIQ and FIQ than healthy children without ADHD. The findings in this study showed that FIQ was approximately 9 points lower in children with ADHD than in healthy children. This is compatible with the results of a previous meta-analytic review, which reported that ADHD groups performed at an average of 9.15 FIQ points lower than the control group [10]. We also found that children with ADHD performed poorly on several subtests of the WISC-III, including Similarities, Vocabulary, and Coding, compared to healthy children. The finding that children with ADHD perform poorly in Coding is consistent with several studies [27-30]. However, there continues to be incongruence in the profile of other WISC-III subtests for children with ADHD. A previous study [9] revealed lower scores in Picture Arrangement, Block Design, and Object Assembly in ADHD groups. But their sample size of 26 control participants and 35 children with ADHD was relatively small. Since their study had limited data and various methodological differences from our study, comparing our results would result in inconsistencies.

The benefits to cognitive function after MPH treatment were noted in many studies [31-34], but there is sparse research and incongruent results in the area of the effect of MPH on subtests of the intelligence test. For example, the ADHD children's improvement on several WISC-III subtests after drug treatment in this study was incompatible with the results of another study that found a positive effect of MPH on the verbal comprehension index [35]. Therefore, the potential influence of MPH on the performance of ADHD children on the WISC-III, except for the IQ scores, needs further research.

There are no recommendations regarding the optimal period for ADHD drug treatment. According to the results of this study, the degree of improvement in ADHD symptom severity was not significantly different relative to the patient's age on receiving MPH treatment. On the other hand, significantly better performance in some subtests and subscales of the WISC-III (such as Similarities, Comprehension, and Object Assembly) was noted in the 6–8 years age group. This suggests that it would be best to start MPH treatment for ADHD at a younger age. However, previous studies have not found that younger age groups obtained more benefits in various aspects than older age groups as a result of MPH treatment. Thus, more research is needed to corroborate this finding and to reach a conclusion.

It has been hypothesized that attention deficit in ADHD children would substantially influence IQ [13], so improving attention would secondarily improve IQ scores. However, this hypothesis was not supported by previous studies, which revealed no significant correlation between the severity of ADHD symptoms and WISC-III IQ scores [36,37]. In addition, the hypothesis that improving attention would secondarily improve IQ scores has also been refuted. Several studies found that improvements in behavioral symptoms had no correlation with changes in cognitive parameters [35,38,39]. This is also consistent with our results that no association was found between improvements in ADHD symptoms and changes in the scores of the WISC-III subtests and subscales (i.e., VCI, POI, FDI, PSI, FIQ, VIQ, and PIQ). It seems that the better performance on intelligence tests after MPH treatment did not result from lessening ADHD symptoms. MPH may possibly produce a different benefit and utilize another mechanism of improving performance on the WISC-III, aside from ameliorating suboptimal test-taking behavior in ADHD children. But MPH may not actually improve construct of IQ as much as it just improves accuracy on the test due to improved attention should be kept in mind.

There are several limitations in this study. First, the sample enrolled in the study did not include all patients with ADHD, so the findings might not be applicable to the entire population of ADHD patients. Second, the sample size in the study was relatively small, particularly for the control group, and some WISC-III subtests (i.e., Symbol Search and Digit Span) were not administered to the control group. Therefore, the statistical power in this study was limited in its ability to detect the changes from pre- to post-treatment and the differences between ADHD and healthy children. Large-scale studies using integrated psychometric measurements are needed. Third, the majority of children with ADHD were male, so the influence of sex could not be ruled out. Fourth, this was not a randomized controlled trial and whether to start MPH treatment or not was based on clinical evaluation and most importantly the opinions of the parents of the ADHD children. Fifth, the interval between the 1^st^ and 2^nd^ administration of the WISC-III was not long enough to ignore the practical effect without controversy. Sixth, other treatments (i.e., cognitive behavioral therapy, parental training, attention training) associated with MPH treatment were often performed in clinical practice, but the interactions and their possible effect on the WISC-III was not followed up in this study.

Although many limitations have been noted, a number of strengths can also be found in this study. First, real-world children with ADHD were sampled and given an appropriate dose of MPH in line with clinical guidelines. Second, the 1^st^ and 2^nd^ administration of the WISC-III was given at least one year apart, thus reducing the possible practical effect to a limited degree [24,40]. Although the sample size of ADHD children with/without MPH treatment was small in this study, the practical effect could not totally explain the improvement in the WISC-III after MPH treatment, because there were no significant changes in the ADHD children who did not receive MPH treatment after one year. Third, all participants were free from medication for at least one week before performing the WISC-III, which would reduce the direct influence of medication on improving test-taking behavior. Thus, the present study is valuable because of its strengths.

## Conclusion

According to the findings in this study, we suggest that long-term MPH treatment may improve neurocognitive profiles, which were shown in the performance of the ADHD children on several subtests and in IQ scores of the WISC-III. And the improvement had no correlation with the decrement of ADHD symptoms and baseline ADHD severity. Starting stimulant treatment at as young an age as possible is advised due to the greater benefits seen in the 6–8 years age group. But more research using a controlled sample to replicate the findings is needed.

## Competing interests

The authors declared that they had no competing interests and received no financial support for this study.

## Authors’ contributions

CST, YSH, KBY, MHT and SMC conceived, designed and participated in the study. CLW and FMH analyzed the data and assisted in result interpretation. All authors read and approved the final manuscript.

## Pre-publication history

The pre-publication history for this paper can be accessed here:

http://www.biomedcentral.com/1471-244X/13/330/prepub
